# Supplementary material for: CD8+ T cells control SIV infection using both cytolytic effects and non-cytolytic suppression of virus production
Source: Nat Commun. 2023 Oct 20;14:6657. doi: 10.1038/s41467-023-42435-8 (PMC10589330; doi:10.1038/s41467-023-42435-8)
Supplement: Supplementary file 1 — Supplementary Information [file 41467_2023_42435_MOESM1_ESM.pdf]

# CD8+ T-cells control SIV infection using both cytolytic effects and non-cytolytic suppression of virus production

**Running head: Cytolytic effects and suppression of virus production by CD8+ T-cells in SIV infection**

## Supplementary Materials

**Authors:** B. B. Policicchio[1#], E. Fabian Cardozo-Ojeda[2#], C. Xu[3], D. Ma[3], T. He[3], K. Raehtz[4], R. Sivanandham[3], A. Kleinman[4], A. S. Perelson[5], C. Apetrei[1,4], I. Pandrea[1,3], R. M. Ribeiro[5,6]

**Affiliations:**
[1]Department of Infectious Diseases and Immunology, Graduate School of Public Health, University of Pittsburgh, Pittsburgh, PA, 15261 USA;
[2]Vaccine and Infectious Disease Division, Fred Hutchinson Cancer Research Center, Seattle, WA, 98109, USA;
[3]Department of Pathology, School of Medicine, University of Pittsburgh, Pittsburgh, PA, 15261, USA;
[4]Division of Infectious Diseases, Department of Medicine, School of Medicine, University of Pittsburgh, Pittsburgh, PA, 15261, USA;
[5]Theoretical Biology and Biophysics Group, Los Alamos National Laboratory, Los Alamos, NM, 87545, USA;
[6]Laboratório de Biomatemática, Faculdade de Medicina da Universidade de Lisboa, Portugal (previous address).

[#]These authors contributed equally.

## SUPPLEMENTARY NOTE 1

### Model with Viral Dynamics Only

To further help interpret the viral load profiles during $CD8^+$ cell depletion and RAL monotherapy, we also used the original slow and rapid integration virus dynamic model[1] without $CD4^+$ T cell proliferation. In this case, we only fit the model to viral load observation and not to the $Ki67^+CD4^+$ T cell count. Without this assumption, the model in equation (1) in the main manuscript has the form:

$$\begin{aligned}
\frac{dT_I}{dt} &= \lambda - dT_I - \beta T_I V \\
\frac{dI_1}{dt} &= \beta T_I V - k(1-\omega)I_1 - \delta_1 I_1 \\
\frac{dM_1}{dt} &= \beta_m T_M V - k_m(1-\omega)M_1 - \delta_m M_1 \qquad \text{(S1).} \\
\frac{dI_2}{dt} &= k_m(1-\omega)M_1 + k(1-\omega)I_1 - \delta_2 I_2 \\
\frac{dV}{dt} &= pI_2 - cV
\end{aligned}$$

To fit the model in equation S1 to the data, we used nonlinear mixed-effect modeling as described in the main text. Unlike in the main manuscript, here we only modeled the plasma viral load for animal $i$ at time $j$ as $y_{ij} = \log_{10} V(t_j) + \epsilon_V$ with $\epsilon_V \sim \mathcal{N}(0, \sigma_v^2)$ the error for the logged viral load.

We performed the data fitting in two steps, as this allowed better convergence of the parameter estimates. We first fitted the model in equation (S1) to the viral load data from the 8 animals under RAL monotherapy only. With this fit, we estimate parameter distributions for $\delta_1$, $\delta_2$, $\delta_m$ to be used in the second step, we also fixed $\beta=10^{-8}$, as this parameter trades-off with $T_I$ in this simple model (see[1] for model details). Other parameters were fixed as in the main text. In these initial fits, $t = 0$ refers to initiation of RAL treatment. We assumed that the system in (S1) is in steady state before $t = 0$, allowing us to obtain the values of $I_1(0) = \frac{\delta_2 c f_I V(0)}{kp}$, $M_1(0) = \frac{\delta_2 c(1-f_I)V(0)}{k_m p}$, $I_2(0) = \frac{cV(0)}{p}$, $T_I(0) = \frac{I_1(0)}{\beta V(0)}(k+\delta_1)$, $\beta_m T_M(0) = \frac{\delta_m + k_m}{V(0)} M_1(0)$ and $\lambda = \big(d + \beta V(0)\big)T_I(0)$.

In a second step, we performed fits of the model in equation (S1) to the viral load data from all 20 animals simultaneously, fixing the same parameters as before and fixing the distribution of $\delta_1$, $\delta_2$, $\delta_m$, as estimated in the RAL-only fits. We repeat each fit by assuming that CD8$^+$ cell depletion has one or more of the following effects: (i) reduction of the death rate of short-lived infected cells before viral integration ($\delta_1$), (ii) reduction of the death rate of productively infected cells ($\delta_2$), (iii) increasing the viral infectivity rate ($\beta$), or (iv) increasing the virus production rate ($p$). These effects were simulated by changing the corresponding parameters in equation (1) under depletion conditions to $(1-\xi_1)\delta_1$, $(1-\xi_2)\delta_2$, $(1+\xi_3)\beta$ and $(1+\xi_4)p$ then estimating $\xi_i$, one at a time or in combination. We thus have 16 different models (Table S3), from all $\xi_i = 0$ (our original model) to all $\xi_i \neq 0$ (indicating the CD8$^+$ depletion affects each of the four parameters). Depending on the effect or combination of effects in each fit, we estimate the respective reduction or increase in each parameter ($\xi_i$). We fit each CD8-depletion effect model to the whole data set 10 times allowing for random initial guesses of the parameters to be estimated. Each of these times, we estimated the log-likelihood ($\log \mathcal{L}$). For the case with highest likelihood ($\max(\log \mathcal{L})$) from the 10 fits, we then computed the corrected Akaike Information Criteria (AIC) as $AIC = -2\max(\log \mathcal{L}) + 2m + \frac{2m(m+1)}{n-m-1}$, where $m$ is the number of parameters estimated and $n$ de number of data points from all animals[2]. We used AIC to compare models with different CD8$^+$ cell effects.

## SUPPLEMENTARY NOTE 2

### Model Identifiability

To assess the identifiability of our models, we used both an analytical approach[3], which analyzes structural identifiability, and the profile likelihood method,[4-6] which provides information on both structural and practical identifiability. For the former, the idea is to find whether scaling

of the parameters to be fitted and unobserved variables leaves the system invariant, exploring symmetries of the equations that are related to more sophisticated methods based on the theory of Lie groups.[3,4,7]

For the profile likelihood method, we used Monolix[8] with each parameter, in turn, fixed at a given value (iterated over a set of values) and fitting the other parameters to assess the log-likelihood of these fits with one less parameter (the fixed one). The 95% confidence interval for the fixed parameter, based on the chi-squared distribution with one degree of freedom is given by the values of the parameter where the -2 log-likelihood is 3.84 units larger than the minimum.[4]

## SUPPLEMENTARY NOTE 3

### Model with Viral Dynamics Only

We first fitted the model to the data from the group of animals receiving RAL monotherapy only, which has also been done for HIV [9]. Virus kinetics analyses have shown that the different phases of viral decline after initiation of treatment can reveal the kinetics of infected cells [1,9]. Therefore, from these fits we estimated the death rate of infected cells before ($\delta_1$) and after integration ($\delta_2$). These estimates will be used as reference when analyzing the effects of $CD8^+$ cell depletion.

We then fitted the model to the three treatment groups together to see what effect of $CD8^+$ cell depletion best explained all the data. Figure S5 show the model predictions using the estimates of best fits to each group of animals. From the best fit, our model predicts that $CD8^+$ cell depletion affects both the loss rate of infected cells before integration (effect in $\delta_1$) and the rate of virus production (effect in $p$), with negligible effect in viral infectivity ($\beta$) and death rate of productively infected cells ($\delta_2$).

**Model Identifiability**

To test the structural identifiability of our model, we used a scaling method based on the invariance of the equations under transformation of the parameters[3]. In this method, one chooses scaling factors $u_i$ for each parameter and unobserved variable and then equate each term of the equations with and without the scaling factor. Those parameters for which the only scaling factor that satisfies these equations is $u_i=1$ are structurally identifiable (see[3] for details).

As an example, let's analyze the first equation of our model, including the scaling factors.

$$u_T \frac{dT_I}{dt} = \lambda - d u_T T_I - u_\beta \beta V u_T T_I + u_r r u_T T_I \left(1 - \frac{T_I + I_1}{u_K K}\right)$$

Note that we only use the scaling factors for parameters to fit (so, λ and $d$, which are kept constant are not multiplied by these factors) and to non-observed variables (so, $T_I+I_1$ is not multiplied because it is our Ki67 observed quantity). Now, dividing both sides by $u_T$, the resulting right-hand side should be equal to the first equation of our model (in the main text) for $dT_I/dt$. Because the equations must be equal for all values of $V(t)$, $T_I(t)$ and $T_I(t)+I_1(t)$, we can equate each functionally independent term of the equations. Doing this we obtain

$$\begin{aligned}
&\frac{d u_T T_I}{u_T} = dT_I \\
&\frac{u_\beta \beta V u_T T_I}{u_T} = \beta V T \Leftrightarrow u_\beta = 1 \\
&\frac{u_r r u_T T_I}{u_T}\left(1 - \frac{T_I + I_1}{u_K K}\right) = r T_I \left(1 - \frac{T_I + I_1}{K}\right) \Rightarrow u_r = u_K \wedge u_K^2 = u_K \Leftrightarrow u_r = 1 \wedge u_K = 1
\end{aligned} ,$$

where the last result requires some simple algebra. Whenever the equalities are satisfied only for scaling factor, $u_i$, equal to 1, the corresponding parameter is identifiable. For example, if the result of the equality was $u_{v1}u_{v2}=1$, then variables $v_1$ and $v_2$ would not be identifiable.

We proceeded in the same way systematically analyzing the other equations of our model Eq. (1) of the main text, for all fitted parameters and unobserved variables, and found that in all cases the scaling factor $u_i$ was 1. Thus, we conclude that our model is structurally identifiable without the factors for the effects of $CD8^+$ cell depletion. When we add these factors, for example $(1 - \xi_1)\ \delta_1$, we see that solution for the scaled equations can have terms of the form $u_{\delta 1}(1-u_{\xi 1}\xi_1)=1-\xi_1$, which don't have a unique solution $u_{\delta 1}=1$ and $u_{\xi 1}=1$. We note that using mixed effects models adds some subtleties to the analysis of structural identifiability[3], since we are fitting all the subjects at the same time, in this case with two slightly different models, with and without the $\xi_i$ factors. Thus, for example, since $\delta_1$ is identifiable from fitting the RAL only macaques, such that $u_{\delta 1}=1$, then also the $u_{\xi 1}=1$ and we recover identifiability. In fact, this is one of the big advantages of population fitting using mixed-effects models.

Next, we analyzed identifiability using the profile likelihood method, which addresses both structural and practical identifiability[4-6]. The results are presented in figure S7 (for the full model from the main text) and S8 (for the viral load only model from this supplementary text).

We found that all parameters for the best fitting model (in Table 1 of the main text) are identifiable (Figure S7), although for some parameters the fitting is not very stable with wide confidence intervals (e.g., parameter $K$). We also tested parameter $\xi_1$ for the reduction in the death rate of pre-integration infected cells, which came out as a relevant parameter in the top models explaining the full data set (Supplementary Table 1). Although the best fits of the model corresponded to values of $\xi_1$ very close to 1, this parameter is difficult to estimate with a flat profile likelihood, in a way consistent with the structural identifiability analysis mentioned above.

For the simpler model, which we fitted in two stages as described, all the fitted parameters were identifiable including $\xi_1$, although the profile likelihoods still show some instability.

# Supplementary Figures

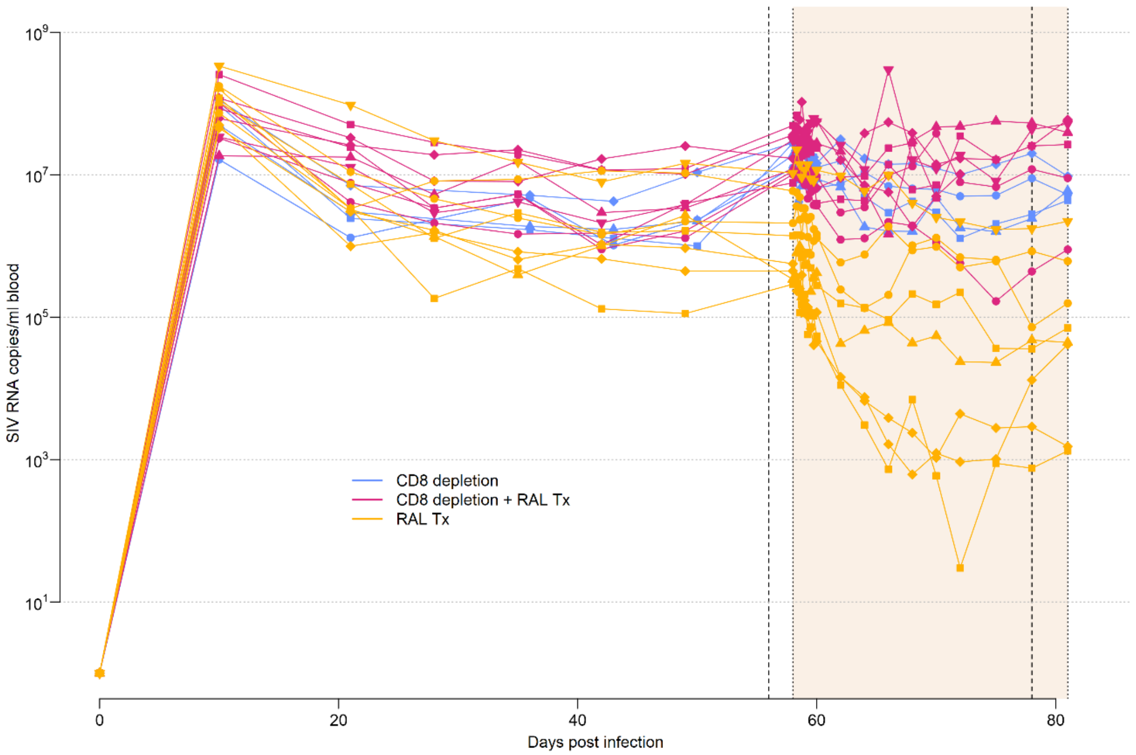


**Supplementary Figure 1. Plasma viral load dynamics in all animals and treatment groups from infection to end of RAL treatment.** Data for the viral load in each individual animal for the three treatment groups in blue for CD8 depletion group (n=4), red for CD8 depletion plus RAL Tx group (n=8), and gold for the RAL Tx group (n=8). Dashed vertical lines represent the times of M-T807R1 administration and the shaded region represents RAL administration, when applicable for each group. Note that the viral load is in quasi-steady state after about 36 days post-infection.

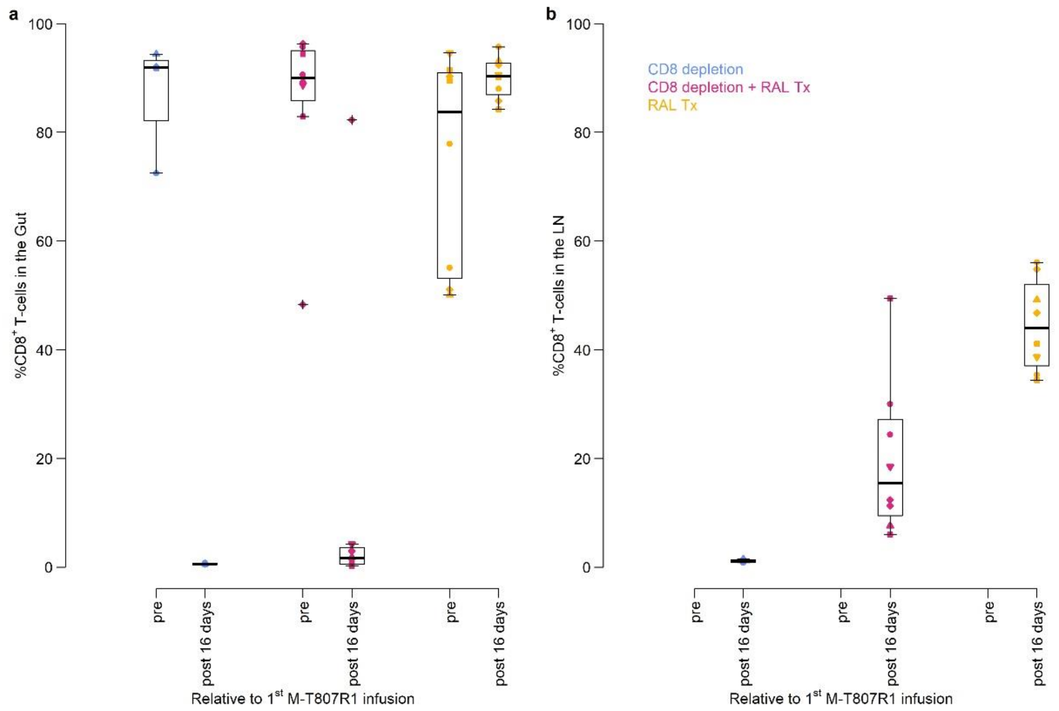


**Supplementary Figure 2. The effect of M-T807R1 administration on gut and lymph node CD8$^+$ T cells.** %CD8$^+$ T cells before the first M-T807R1 administration (after viral load has reached steady state), and 16 days after in **(a)** jejunal biopsy specimens and **(b)** superficial lymph nodes (LNs). For animal health reasons, we did not perform a LN biopsy sample before the CD8-cell depletion. Blue: CD8 depletion group (n=4); red: CD8 depletion plus RAL Tx group (n=8); and gold: RAL Tx group (n=8). Individual data points and box plots are presented. Box plots represent the 25$^{th}$ and 75$^{th}$ percentiles (bottom and top edge of the box), the median (line across the box), whiskers extending from the edge of the box to the smallest (bottom) or largest (top) value no further than 1.5 times the interquartile range from the box's edges, and extreme values beyond that shown as crosses.

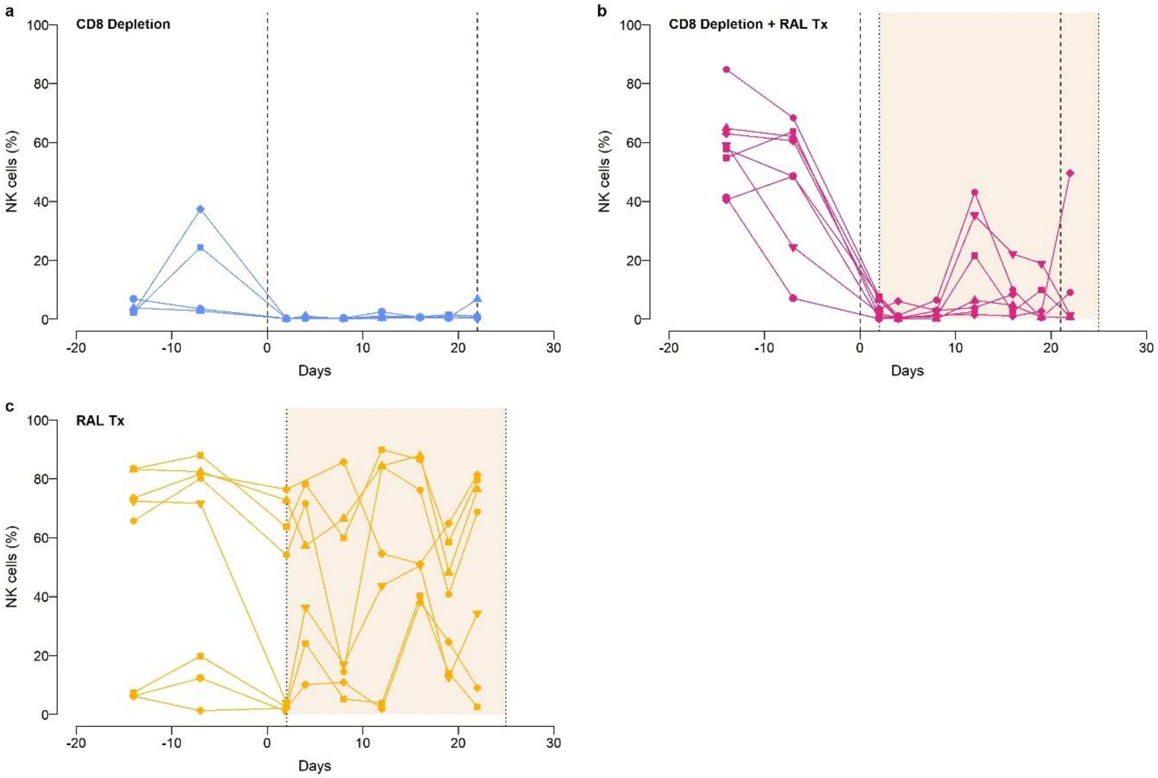


**Supplementary Figure 3. The effect of M-T807R1 administrations and RAL monotherapy on peripheral NK cells.** The percentage of NK cells ($NKG2A^{+}$) is shown for each animal in the CD8 depletion group (a, n=4); the CD8 depletion plus RAL Tx group (b, n=8); and the RAL Tx group (c, n=8). Dashed vertical lines represent the times of M-T807R1 administration and shaded regions represent RAL monotherapy.

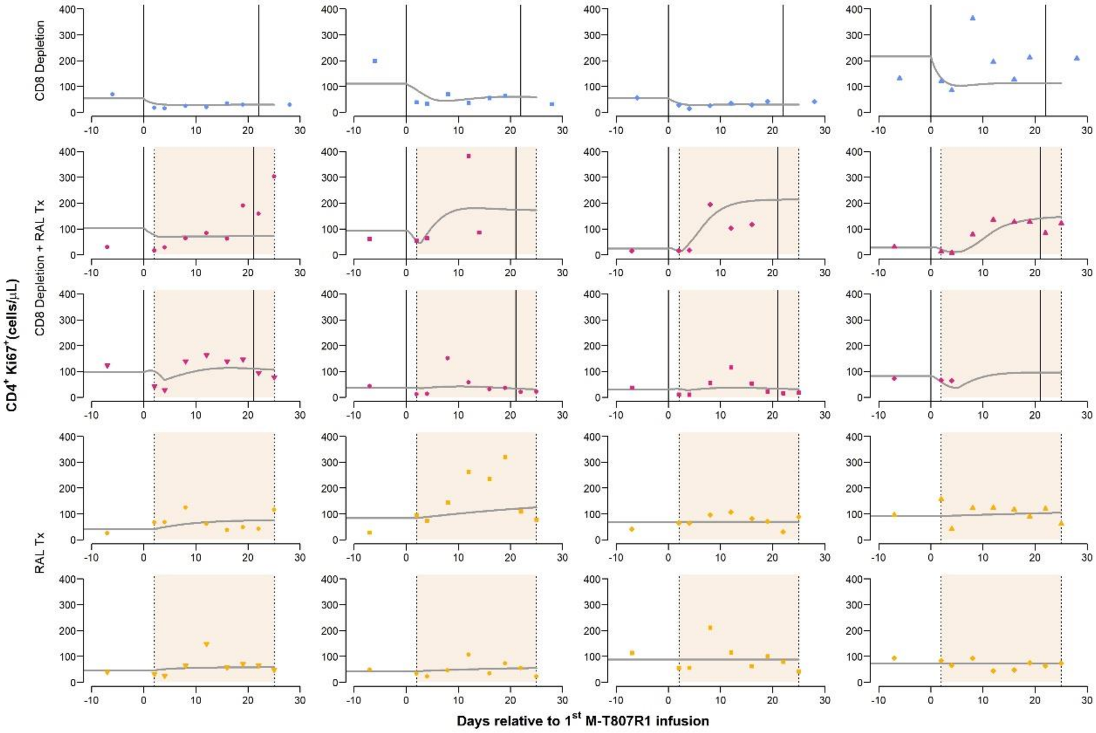


**Supplementary Figure 4. Fits of the best model to the Ki67$^+$ CD4$^+$ T-cell count data.** Each panel presents the dynamics of Ki67$^+$ CD4$^+$ T cells (symbols) of an individual macaque for the three study groups: CD8 depletion group (top row); CD8 depletion plus RAL Tx group (2nd and 3$^{rd}$ rows); and RAL Tx group (bottom two rows). Vertical-solid lines represent the times of M-T807R1 administration, and the shaded regions represent the time of RAL monotherapy. Gray solid lines in each panel represent the model fit using individual parameter estimates in Table S2.

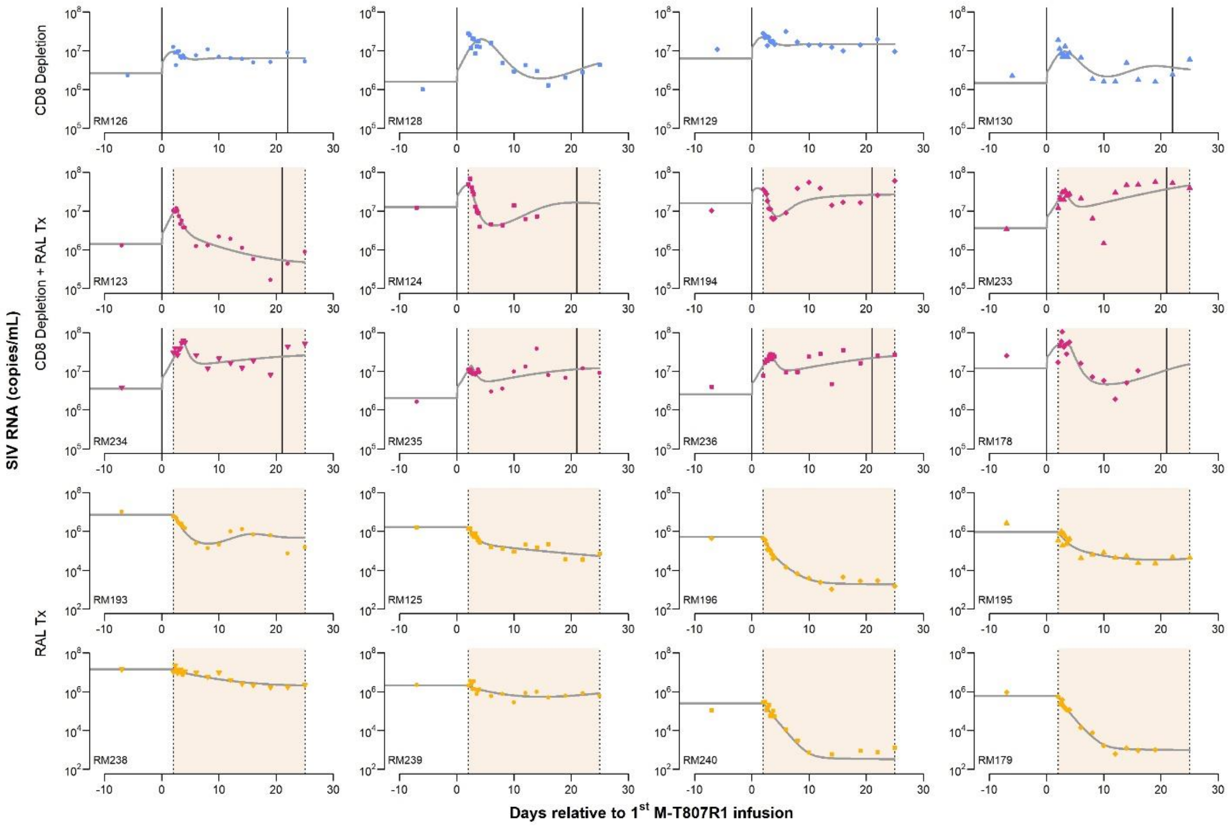


**Supplementary Figure 5. Virus dynamics fits from the alternative model of viral dynamics developed to analyze only the observed VL data.** Each panel presents the viral load dynamics (symbols) of an individual macaque for the three study groups: CD8 depletion group (top row), CD8 depletion plus RAL Tx group (2nd and 3rd rows), and RAL Tx group (bottom two rows). Vertical-solid lines represent the times of M-T807R1 administration and shaded regions represent the time of RAL monotherapy. Gray solid lines represent the model fits to the individual macaques.

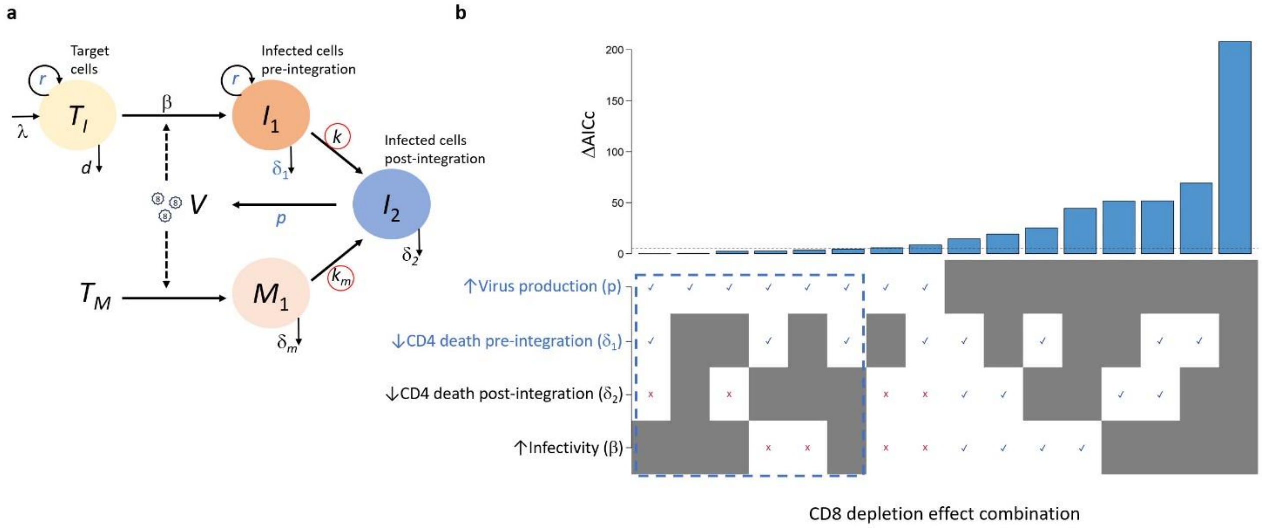


**Supplementary Figure 6. Mathematical model schematics and CD8⁺ cell effects modeled when fitting the simplified model to the viral load only. a.** Diagram describing the viral dynamics mathematical model. $T_I$ represents target cells that after infection will be short-lived pre-integration infected cells $I_1$. $T_M$ represents a constant level of target cells that after infection become long-lived pre-integration infected cells $M_1$. $I_2$ represents infected cells after SIV DNA integration that produce virus $V$. Parameters inside red circles, represent the parameters affected by raltegravir. Effects/parameters in blue are those selected by the model selection procedure. Other details are described in Methods. **b.** Difference in the corrected Akaike Information Criteria (ΔAICc) of each model instance in relation to the one with lowest AICc. Models with ΔAICc<5 represent the most parsimonious models (dashed blue square below the plot). The 16 model instances (represented by the bars in the plot and the columns in the table below the plot) codify a combination of effects that may occur under CD8⁺ cell depletion: (i) increase in the viral production rate, (ii) reduction in the infected cell death rate pre-integration, (iii) reduction in the productively infected cell death rate, or (iv) increase in the viral infection rate. Gray patches in the table represent effects that *were not* tested in that model instance (in each column). Black check marks indicate that the corresponding effect was significant in that model and red cross symbols represent effects that were tested but not significant in that model instance.

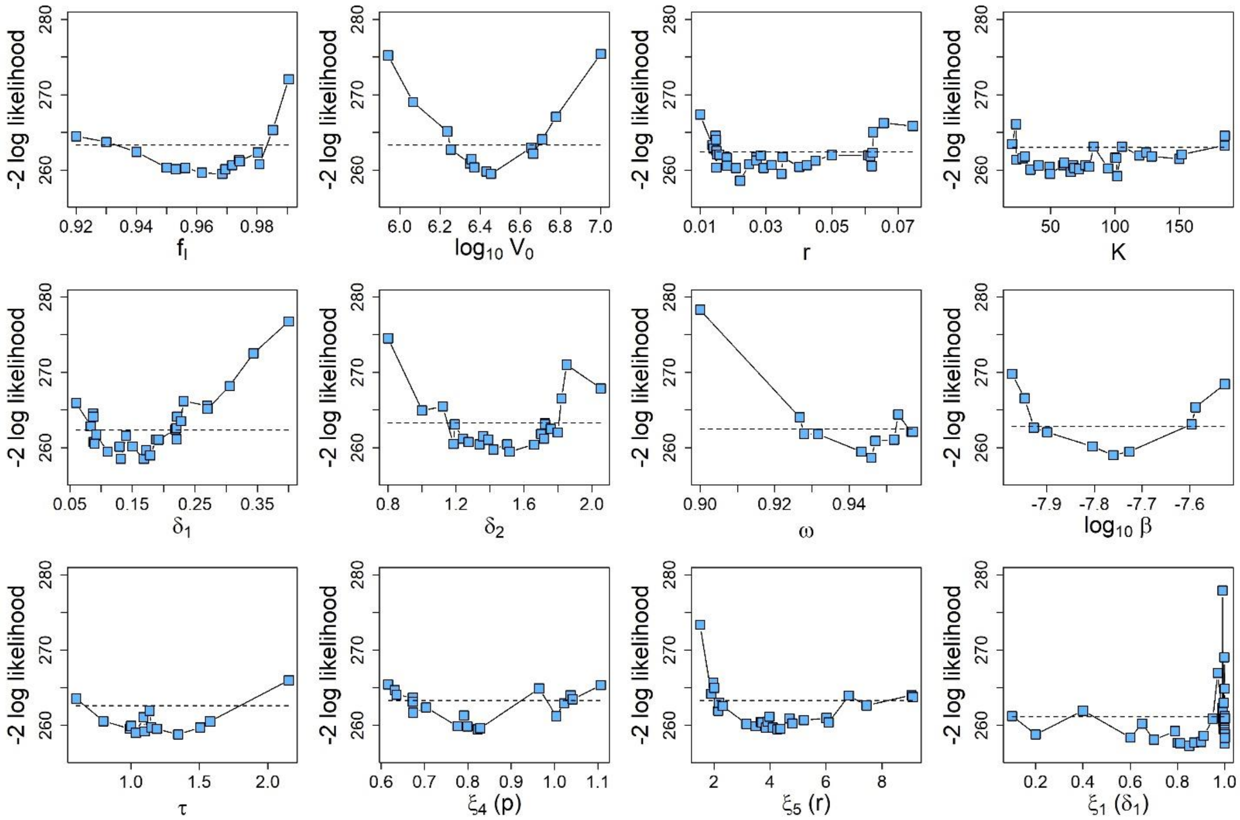


**Supplementary Figure S7. Likelihood profiles for the parameters fitted in the best full model (main text and Tables 1 and S1).** The full model was re-fitted keeping each parameter (each panel) at the values indicated in the x-axis and the -2 log-likelihood is plotted on the y-axis. The dashed line indicates the minimum -2 log-likelihood plus 3.84 which is the threshold for 95% confidence interval. The last panel for $\xi_1$ is for the second-best model in Table S1 to show the profile likelihood for this factor.

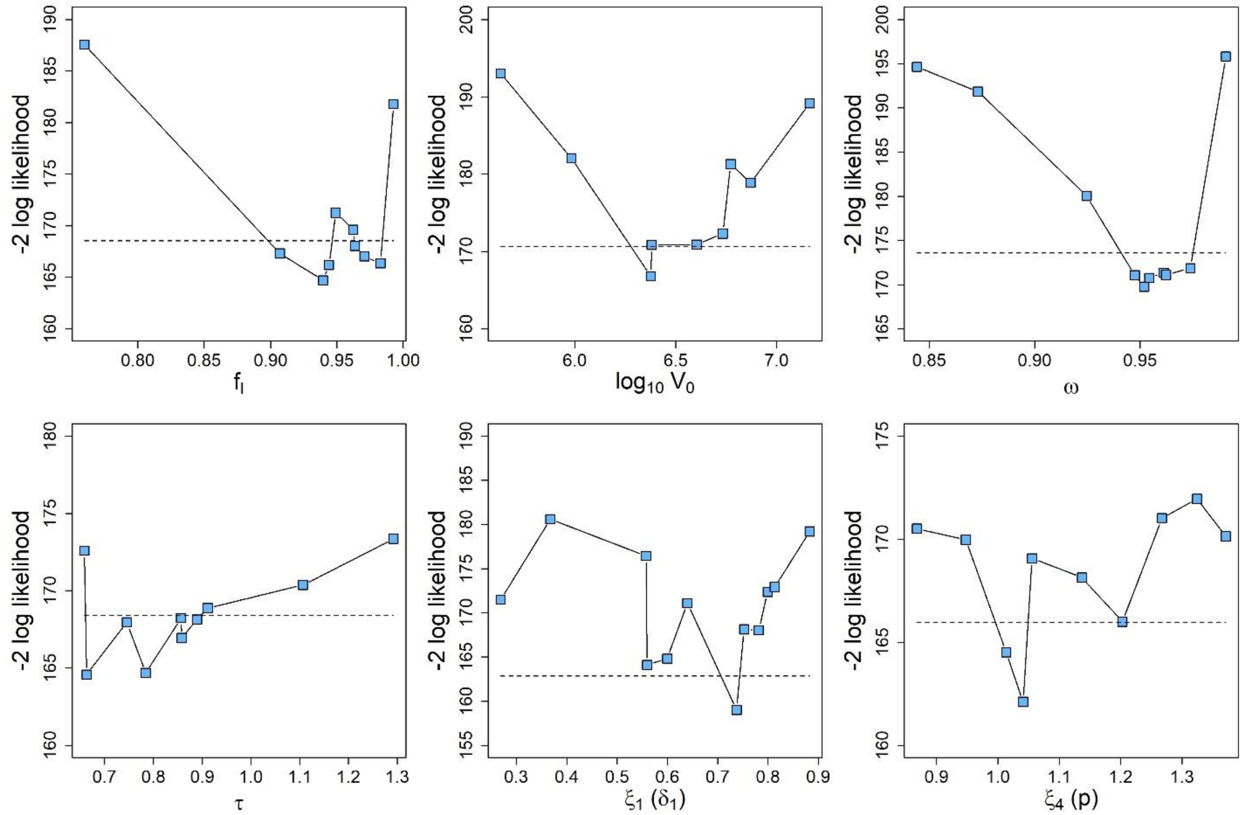


**Supplementary Figure S8. Likelihood profiles for the parameters fitted in the best model analyzing viral load only (supplementary note 1 and Tables S3).** The viral load-only model was refitted keeping each parameter (each panel) at the values indicated in the x-axis and the -2 log-likelihood is plotted on the y-axis. The dashed line indicates the minimum -2 log-likelihood plus 3.84 which is the threshold for 95% confidence interval. As indicated in the supplementary note, we fixed some of the parameters in these fits and these are not represented here.

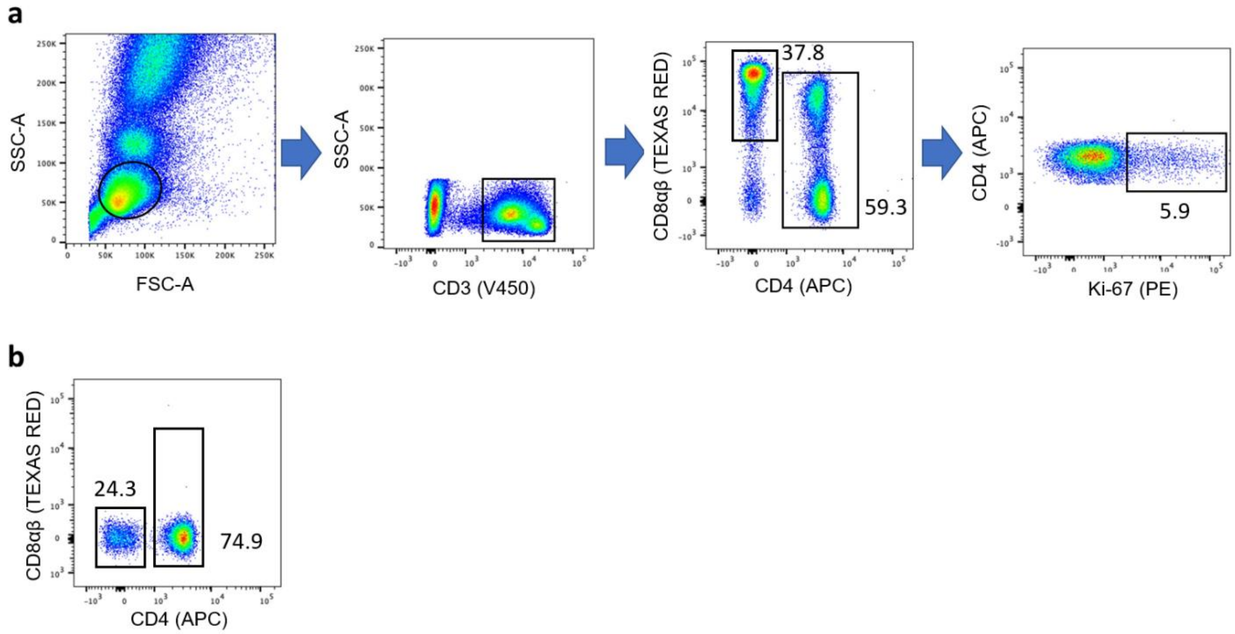


**Supplementary Figure S9. Example of gating strategy for (a) Ki67+ CD4+ cells and (b) an example of CD8 depletion.** Cells were gated first on the lymphocyte population. We then selected the CD3pos T cells on which we gated the CD4pos and CD8pos T cells. Ki-67 was then gated on CD4pos T cells.

# Supplementary Tables

**Supplementary Table 1. Difference in the corrected Akaike Information Criteria (ΔAICc) for the 32 model instances codifying different combinations of CD8⁺ cell depletion effects.** ΔAICc compares the AICc of a model instance with respect with the one with lowest AICc. Models with ΔAICc<5 represent the most parsimonious models (dashed blue square in the CD8⁺ cell effect combinations). Model instances codify one of a combination of effects of CD8⁺ cell depletion: (i) reduction of the death rate of short-lived infected cells before viral integration ($\delta_1$), (ii) reduction of the death rate of productively infected cells ($\delta_2$), (iii) increase in the viral infectivity rate ($\beta$), or (iv) increase in the virus production rate ($p$). We also allow for an effect of depletion increasing the CD4⁺ T cell proliferation rate ($r$). These effects were simulated by changing the corresponding parameters in equation (1) under depletion conditions to $(1-\xi_1)\delta_1$, $(1-\xi_2)\delta_2$, $(1+\xi_3)\beta$, $(1+\xi_4)p$ and $(1+\xi_5)r$. Each row is a model instance. Red values per row represent $\xi_i$ <u>estimates</u> not significantly different from zero. Gray boxes with zeros represent fixed values of $\xi_i$, when the respective CD8⁺ cell effect <u>was not considered</u> in that model instance. This table corresponds to the data presented in Figure 5b.

| | Parameters affected by CD8 depletion | | | | | #Parameters | ΔAICc |
|---|---|---|---|---|---|---|---|
| | $r$ | $p$ | $\delta_1$ | $\delta_2$ | $\beta$ | | |
| | $\xi_5$ | $\xi_4$ | $\xi_1$ | $\xi_2$ | $\xi_3$ | | |
| CD8 effect combination | 3.86 | 0.84 | 0 | 0 | 0 | 20 | 0.00 |
| | 4.03 | 0.83 | 1.00 | 0 | 0 | 21 | 0.45 |
| | 3.59 | 0.84 | 0 | 2.22E-16 | 0 | 21 | 1.44 |
| | 3.50 | 0.84 | 1.00 | 2.22E-16 | 0 | 22 | 2.11 |
| | 3.85 | 0.84 | 0 | 0 | 2.06E-17 | 21 | 2.16 |
| | 3.99 | 0.88 | 1.00 | 0 | 1.54E-19 | 22 | 2.39 |
| | 3.92 | 0.83 | 0 | 2.22E-16 | 1.09E-16 | 22 | 3.96 |
| | 4.55 | 0.87 | 1.00 | 6.91E-14 | 7.40E-16 | 23 | 4.60 |
| | 6.05 | 0 | 1.00 | 0 | 1.34 | 21 | 7.55 |
| | 5.87 | 0 | 0 | 0 | 1.42 | 20 | 7.77 |
| | 7.26 | 0 | 1.00 | 0.29 | 0.71 | 22 | 7.89 |

| | | | | | | | |
|---|---|---|---|---|---|---|---|
| | 6.61 | 0 | 0 | 0.22 | 0.88 | 21 | 10.15 |
| | 7.91 | 0 | 1.00 | 0.54 | 0 | 21 | 11.43 |
| | 5.17 | 0 | 0 | 0.51 | 0 | 20 | 13.22 |
| | 0 | 0 | 1.00 | 0.39 | 0 | 20 | 15.86 |
| | 0 | 0.53 | 1.00 | 2.77E-16 | 1.10E-15 | 22 | 18.61 |
| | 0 | 0.56 | 1.00 | 0 | 1.13E-16 | 21 | 19.70 |
| | 0 | 0.51 | 1.00 | 2.79E-16 | 0 | 21 | 22.66 |
| | 0 | 0 | 1.00 | 0.35 | 1.09E-16 | 21 | 22.78 |
| | 0 | 0.65 | 1.00 | 0 | 0 | 20 | 24.98 |
| | 0 | 0 | 1.00 | 0 | 0.68 | 20 | 29.71 |
| | 0 | 0.77 | 0 | 0 | 0 | 19 | 40.42 |
| | 0 | 0.78 | 0 | 5.14E-15 | 4.07E-18 | 21 | 42.34 |
| | 0 | 0.77 | 0 | 0 | 5.01E-62 | 20 | 42.71 |
| | 0 | 0 | 0 | 0.50 | 0 | 19 | 43.70 |
| | 0 | 0 | 0 | 0.50 | 2.51E-26 | 20 | 43.88 |
| | 0 | 0 | 0 | 0.49 | 0 | 20 | 45.21 |
| | 2.53 | 0 | 1.00 | 0 | 0 | 20 | 45.97 |
| | 0 | 0 | 0 | 0 | 1.23 | 19 | 60.57 |
| | 0 | 0 | 1.00 | 0 | 0 | 19 | 84.20 |
| | 6.87 | 0 | 0 | 0 | 0 | 19 | 116.94 |
| | 0 | 0 | 0 | 0 | 0 | 18 | 201.44 |

Note: The AICc value for the best model is 301.6.

**Supplementary Table 2. Individual parameter estimates for the model in which CD8⁺ cells affect death rate of infected cells prior to integration, CD4⁺ T cell proliferation and virus production.** For all animals the population estimates for the CD8+ cell effects were: $\xi_4 = 0.84$ and $\xi_5 = 3.9$.

| | ID | $f_I$ | $\log_{10} V_0$ | $r$ | $K$ | $\delta_1$ | $\delta_2$ | $\delta_m$ | $\omega$ | $\log_{10}\beta$ | $p$ | $\tau$ |
|---|---|---|---|---|---|---|---|---|---|---|---|---|
| **CD8 depletion** | **RM126** | 0.96 | 6.4 | 0.04 | 16.5 | 0.14 | 1.3 | 0.02 | - | -7.8 | 39,546 | - |
| | **RM128** | 0.97 | 6.2 | 0.01 | 29.4 | 0.15 | 2.6 | 0.02 | - | -7.9 | 41,384 | - |
| | **RM129** | 0.96 | 6.8 | 0.03 | 17.4 | 0.14 | 1.3 | 0.02 | - | -7.8 | 38,948 | - |
| | **RM130** | 0.97 | 6.3 | 0.01 | 31.2 | 0.15 | 3.7 | 0.02 | - | -7.8 | 24,722 | - |
| **CD8 depletion & RAL** | **RM123** | 0.96 | 6.2 | 0.01 | 137.4 | 0.25 | 1.7 | 0.02 | 0.95 | -7.7 | 28,935 | 0.6 |
| | **RM124** | 0.96 | 7.2 | 0.03 | 154.6 | 0.14 | 2.3 | 0.02 | 0.94 | -7.6 | 28,261 | 0.7 |
| | **RM194** | 0.96 | 7.1 | 0.06 | 881.6 | 0.13 | 2.0 | 0.02 | 0.95 | -7.1 | 33,596 | 0.5 |
| | **RM233** | 0.96 | 6.8 | 0.07 | 832.8 | 0.14 | 1.4 | 0.02 | 0.93 | -7.4 | 31,457 | 4.0 |
| | **RM234** | 0.96 | 6.6 | 0.04 | 678.6 | 0.13 | 1.2 | 0.02 | 0.94 | -8.0 | 31,246 | 2.0 |
| | **RM235** | 0.97 | 6.2 | 0.03 | 261.4 | 0.13 | 1.0 | 0.02 | 0.93 | -7.7 | 36,680 | 0.8 |
| | **RM236** | 0.96 | 6.4 | 0.04 | 305.7 | 0.12 | 0.9 | 0.02 | 0.94 | -7.8 | 53,754 | 1.4 |
| | **RM178** | 0.96 | 7.1 | 0.02 | 174.6 | 0.14 | 1.1 | 0.02 | 0.94 | -7.9 | 29,163 | 2.9 |
| **RAL** | **RM193** | 0.96 | 6.9 | 0.02 | 15.5 | 0.13 | 1.4 | 0.02 | 0.95 | -7.7 | 43,179 | 1.1 |
| | **RM125** | 0.97 | 6.3 | 0.02 | 137.1 | 0.15 | 1.6 | 0.02 | 0.94 | -7.8 | 28,317 | 1.1 |
| | **RM196** | 0.95 | 5.8 | 0.03 | 3.2 | 0.15 | 2.3 | 0.02 | 0.94 | -7.7 | 44,291 | 1.1 |
| | **RM195** | 0.96 | 6.0 | 0.02 | 171.7 | 0.13 | 1.1 | 0.02 | 0.95 | -8.0 | 27,050 | 1.1 |
| | **RM238** | 0.97 | 7.2 | 0.03 | 3.0 | 0.14 | 1.0 | 0.02 | 0.66 | -7.8 | 38,789 | 1.1 |
| | **RM239** | 0.96 | 6.3 | 0.02 | 12.8 | 0.16 | 1.4 | 0.02 | 0.67 | -7.7 | 41,336 | 1.1 |
| | **RM240** | 0.98 | 5.4 | 0.03 | 1.0 | 0.14 | 1.0 | 0.02 | 0.94 | -7.8 | 35,313 | 1.2 |
| | **RM179** | 0.97 | 5.8 | 0.03 | 2.3 | 0.14 | 1.5 | 0.02 | 0.94 | -7.8 | 38,345 | 1.1 |

**Supplementary Table 3. Difference in the corrected Akaike Information Criteria (ΔAICc) for the 16 model instances codifying different combinations of CD8+ cell depletion effects in the alternative model fitting only to viral loads.** ΔAICc compares the AICc of a model instance with respect with the one with lowest AICc. Models with ΔAICc<5 represent the most parsimonious models (dashed blue square in the CD8+ cell effect combinations). Model instances codify one a combination of effects that CD8+ cells: (i) reduction of the death rate of short-lived infected cells before viral integration ($\delta_1$), (ii) reduction of the death rate of productively infected cells ($\delta_2$), (iii) increase in the viral infectivity rate ($\beta$), or (iv) increase in the virus production rate ($p$). These effects were simulated by changing the corresponding parameters in equation (S1) under depletion conditions to $(1-\xi_1)\delta_1$, $(1-\xi_2)\delta_2$, $(1+\xi_3)\beta$ and $(1+\xi_4)p$. Each row is a model instance. Each row is a model instance. Red values per row represent $\xi_i$ estimates not significantly different from zero. Gray boxes represent fixed values of $\xi_i$, when the respective CD8+ cell effect was not considered in that instance. This table corresponds to the data presented in Supplementary Figure 6b.

| | Parameter affected by CD8 depletion | | | | #Parameters | ΔAICc |
|---|---|---|---|---|---|---|
| | $p$ | $\delta_1$ | $\delta_2$ | $\beta$ | | |
| | $\xi_4$ | $\xi_1$ | $\xi_2$ | $\xi_3$ | | |
| CD8 effect combination | 1.05 | 0.74 | 1.59E-06 | 0 | 12 | 0.00 |
| | 1.06 | 0 | 0 | 0 | 10 | 0.16 |
| | 1.06 | 0 | 1.16E-05 | 0 | 11 | 2.22 |
| | 0.96 | 0.71 | 0 | 1.78E-04 | 12 | 2.50 |
| | 1.05 | 0 | 0 | 6.17E-08 | 11 | 3.34 |
| | 1.01 | 0.75 | 0 | 0 | 11 | 4.44 |
| | 1.04 | 0 | 6.72E-06 | 5.79E-08 | 12 | 5.75 |
| | 0.95 | 0.77 | 3.91E-05 | 2.91E-07 | 13 | 8.54 |
| | 0 | 0.84 | 0.30 | 0.97 | 12 | 14.49 |
| | 0 | 0 | 0.39 | 1.04 | 11 | 19.10 |
| | 0 | 0.93 | 0 | 1.26 | 11 | 25.26 |
| | 0 | 0 | 0 | 1.38 | 10 | 44.44 |
| | 0 | 0 | 0.59 | 0 | 10 | 51.58 |
| | 0 | 0.91 | 0.46 | 0 | 11 | 51.70 |
| | 0 | 0.98 | 0 | 0 | 10 | 69.07 |
| | 0 | 0 | 0 | 0 | 8 | 208.06 |

Note: The AICc value for the best model is 184.2.

# References

1. Cardozo, E.F., *et al.* Treatment with integrase inhibitor suggests a new interpretation of HIV RNA decay curves that reveals a subset of cells with slow integration. *PLoS Pathog* **13**, e1006478 (2017).
2. Burnham, K. & Anderson, D. *Model Selection and Multimodel Inference*, (Springer, New York, New York, 2004).
3. Castro, M. & de Boer, R.J. Testing structural identifiability by a simple scaling method. *PLoS Comput Biol* **16**, e1008248 (2020).
4. Maiwald, T., *et al.* Driving the Model to Its Limit: Profile Likelihood Based Model Reduction. *PLoS ONE* **11**, e0162366 (2016).
5. Mitra, E.D. & Hlavacek, W.S. Parameter Estimation and Uncertainty Quantification for Systems Biology Models. *Curr Opin Syst Biol* **18**, 9-18 (2019).
6. Raue, A., *et al.* Structural and practical identifiability analysis of partially observed dynamical models by exploiting the profile likelihood. *Bioinformatics* **25**, 1923-1929 (2009).
7. Merkt, B., Timmer, J. & Kaschek, D. Higher-order Lie symmetries in identifiability and predictability analysis of dynamic models. *Phys Rev E Stat Nonlin Soft Matter Phys* **92**, 012920 (2015).
8. Lavielle, M. *Mixed Effects Models for the Population Approach: Models, Tasks, Methods and Tools*, (Chapman and Hall/CRC, Boca Raton, 2014).
9. Andrade, A., *et al.* Early HIV RNA decay during raltegravir-containing regimens exhibits two distinct subphases (1a and 1b). *AIDS (London, England)* **29**, 2419-2426 (2015).
