# Supplementary material for: CD8+ T cells control SIV infection using both cytolytic effects and non-cytolytic suppression of virus production
Source: Nat Commun. 2023 Oct 20;14:6657. doi: 10.1038/s41467-023-42435-8 (PMC10589330; doi:10.1038/s41467-023-42435-8)
Supplement: Supplementary file 3 — Reporting Summary [file 41467_2023_42435_MOESM3_ESM.pdf]

nature portfolio

Corresponding author(s): Ribeiro, RM

Last updated by author(s): Aug 31, 2023

Reporting Summary

Nature Portfolio wishes to improve the reproducibility of the work that we publish. This form provides structure for consistency and transparency in reporting. For further information on Nature Portfolio policies, see our Editorial Policies and the Editorial Policy Checklist.

Statistics

For all statistical analyses, confirm that the following items are present in the figure legend, table legend, main text, or Methods section.

n/a	Confirmed	
☐	☒	The exact sample size (*n*) for each experimental group/condition, given as a discrete number and unit of measurement
☐	☒	A statement on whether measurements were taken from distinct samples or whether the same sample was measured repeatedly
☐	☒	The statistical test(s) used AND whether they are one- or two-sided<br>*Only common tests should be described solely by name; describe more complex techniques in the Methods section.*
☒	☐	A description of all covariates tested
☐	☒	A description of any assumptions or corrections, such as tests of normality and adjustment for multiple comparisons
☐	☒	A full description of the statistical parameters including central tendency (e.g. means) or other basic estimates (e.g. regression coefficient) AND variation (e.g. standard deviation) or associated estimates of uncertainty (e.g. confidence intervals)
☐	☒	For null hypothesis testing, the test statistic (e.g. *F*, *t*, *r*) with confidence intervals, effect sizes, degrees of freedom and *P* value noted<br>*Give P values as exact values whenever suitable.*
☒	☐	For Bayesian analysis, information on the choice of priors and Markov chain Monte Carlo settings
☒	☐	For hierarchical and complex designs, identification of the appropriate level for tests and full reporting of outcomes
☒	☐	Estimates of effect sizes (e.g. Cohen's *d*, Pearson's *r*), indicating how they were calculated

Our web collection on statistics for biologists contains articles on many of the points above.

Software and code

Policy information about availability of computer code

Data collection	BD FACSDiva v8.01 for the BD LSR II flow cytometer. Applied Biosystems SDSv2.4.1 software for the Applied Biosystems 7900HT real-time PCR machine.
Data analysis	FLowJo v10.4 and v10.7.1. Monolix versions 2020R1, 2021R1, R v 4.2.0, with package lmerTest v 3.1-3.

For manuscripts utilizing custom algorithms or software that are central to the research but not yet described in published literature, software must be made available to editors and reviewers. We strongly encourage code deposition in a community repository (e.g. GitHub). See the Nature Portfolio guidelines for submitting code & software for further information.

Data

Policy information about availability of data

All manuscripts must include a data availability statement. This statement should provide the following information, where applicable:

- Accession codes, unique identifiers, or web links for publicly available datasets
- A description of any restrictions on data availability
- For clinical datasets or third party data, please ensure that the statement adheres to our policy

The raw data for all graphs generated in this study are provided in the Supplementary Information and Source Data file. Source data are provided with this paper.

Research involving human participants, their data, or biological material

Policy information about studies with human participants or human data. See also policy information about sex, gender (identity/presentation), and sexual orientation and race, ethnicity and racism.

Reporting on sex and gender	NA
Reporting on race, ethnicity, or other socially relevant groupings	NA
Population characteristics	NA
Recruitment	NA
Ethics oversight	NA

Note that full information on the approval of the study protocol must also be provided in the manuscript.

Field-specific reporting

Please select the one below that is the best fit for your research. If you are not sure, read the appropriate sections before making your selection.

- [x] Life sciences
- [ ] Behavioural & social sciences
- [ ] Ecological, evolutionary & environmental sciences

For a reference copy of the document with all sections, see nature.com/documents/nr-reporting-summary-flat.pdf

Life sciences study design

All studies must disclose on these points even when the disclosure is negative.

Sample size	For direct comparisons, eight animals each for the two RAL-treatment groups, based on previous data on SIV-infected ART-treated rhesus macaques, allows detection of a difference in the level of plasma SIV RNA with an effect size of 2, at alpha=0.05 significance level with a power of at least 0.9. For mixed-effects tests and mixed-effects population fittings no formal sample size determination was performed. This modeling approach has more power to detect differences than simper statistical tests since it uses time courses.
Data exclusions	No data exclusions.
Replication	All animals and measurements taken are in the manuscript. Replication was over the 20 animals in the study, and all animals planned were included in the study. All replication experiments were successful - generating the data presented.
Randomization	Animals were sequentially attributed to each of the study groups, with the control untreated group being the first studied.
Blinding	Blinding of the investigators was not possible as they were responsible for the distribution of compounds for administration and treatment allocation, and definition of the model fitting. Initial data analyses were blinded, but the data dynamics clearly differentiated the groups, so final data analyses and fitting were not blinded.

Reporting for specific materials, systems and methods

We require information from authors about some types of materials, experimental systems and methods used in many studies. Here, indicate whether each material, system or method listed is relevant to your study. If you are not sure if a list item applies to your research, read the appropriate section before selecting a response.

Materials & experimental systems

n/a	Involved in the study	
[ ]	[x]	Antibodies
[x]	[ ]	Eukaryotic cell lines
[x]	[ ]	Palaeontology and archaeology
[ ]	[x]	Animals and other organisms
[x]	[ ]	Clinical data
[x]	[ ]	Dual use research of concern
[x]	[ ]	Plants

Methods

n/a	Involved in the study	
[x]	[ ]	ChIP-seq
[ ]	[x]	Flow cytometry
[x]	[ ]	MRI-based neuroimaging

Antibodies

Antibodies used	All antibodies were used as per manufacturer recommendation. From BD Biosciences: CD4 (APC, clone L200, cat# 551980, 2.5ul),

	CD8 (PE-CF594, clone RPA-T8, cat#: 562282, 3ul), CD3 (V450, clone SP34-2, cat#: 560351, 2ul), CD45 (PerCP, clone D058-1283, cat#: 558411, 3ul), Ki-67 (PE, clone B56, cat#: 556027, 20ul). From Beckman Coulter Life Sciences NKG2A (PE, clone Z199, cat#: IM3291U, 5ul). NIH Nonhuman Primate Reagent Resource M-T807R1 (clone ID MT807R1, Cat# PR-0817, RRID:AB2716320)
Validation	All antibodies (except NKG2A) were validated for usage in rhesus macaques by the NIH (as indicated in www.nhpreagents.org/ReactivityDatabase), and in previous nonhuman primate studies (see ref. 18-26, 29). NKG2A was validated for cross-reactivity in rhesus macaques by the manufacturer (https://www.beckman.com/reagents/coulter-flow-cytometry/antibodies-and-kits/single-color-antibodies/cd159a/im3291u).

Animals and other research organisms

Policy information about studies involving animals; ARRIVE guidelines recommended for reporting animal research, and Sex and Gender in Research

Laboratory animals	Indian-origin rhesus macaques (Macaca mulatta), all males, 5-7 years of age.
Wild animals	This study did not involve wild animals.
Reporting on sex	Sex of the animals is reported. Sex was not considered in the study design, and the analyses was not done by sex. The number of animals used did not allow inclusion of sex as a biological variable.
Field-collected samples	This study did not involve field collected samples.
Ethics oversight	Animal experiments were approved by the University of Pittsburgh Institutional Animal Care and Use Committee (IACUC), protocol #16058287.

Note that full information on the approval of the study protocol must also be provided in the manuscript.

Flow Cytometry

Plots

Confirm that:

- [x] The axis labels state the marker and fluorochrome used (e.g. CD4-FITC).
- [x] The axis scales are clearly visible. Include numbers along axes only for bottom left plot of group (a 'group' is an analysis of identical markers).
- [x] All plots are contour plots with outliers or pseudocolor plots.
- [x] A numerical value for number of cells or percentage (with statistics) is provided.

Methodology

Sample preparation	Whole blood was stained with fluorescently-labeled antibodies to identify various cell populations. Briefly, antibodies were added to whole blood and washed several times prior to being acquired using a BD LSRII flow cytometer. Two-step TruCount (BD Bioscience) technique was used with whole blood following provided instructions.
Instrument	BD Biosciences LSR II flow cytometer
Software	BD FACSDiva v8.01 for sample analyses, and FlowJo v.10.4 and 10.7.1 for data analyses.
Cell population abundance	FACS sorting of cellular subsets was not performed in this study.
Gating strategy	CD4+ and CD8+ T cell percentages were obtained by first gating on lymphocytes, then on CD3+ T cells. T cell proliferation status (Ki-67) was assessed by gating on lymphocytes, then on CD3+ T cells, and finally on CD4+ CD3+ T cells. NKG2A was used to identify circulating NK cells, by gating first on lymphocytes and then on CD3neg immune cell population.

- [x] Tick this box to confirm that a figure exemplifying the gating strategy is provided in the Supplementary Information.
